# IMPLEMENTING REABLEMENT IN COMMUNITY CARE: HOW DO WE SUCCEED?

**DOI:** 10.1093/geroni/igad104.2249

**Published:** 2023-12-21

**Authors:** Ines Mouchaers, Lise Buma, Hilde Verbeek, Sandra Zwakhalen, Ellen Vlaeyen, Geert Goderis, Silke Metzelthin

**Affiliations:** Maastricht University, Maastricht, Limburg, Netherlands; Maastricht University, Maastricht, Limburg, Netherlands; Maastricht University, Maastricht, Limburg, Netherlands; Maastricht University, Maastricht, Limburg, Netherlands; KU Leuven, Leuven, Vlaams-Brabant, Belgium; KU Leuven, Leuven, Vlaams-Brabant, Belgium; Maastricht University, Care and Public Health Research Institute, Maastricht, Limburg, Netherlands

## Abstract

In the Netherlands, reablement is high on the agenda for inclusion in future health care policy, resulting in more and more care providers wanting to implement reablement into their everyday practice. So far, it has been implemented, resourced and organized in multiple ways. To support the implementation on an (inter)national level, insight is needed into what preconditions and influencing factors are regarding the successful implementation of reablement. Within a qualitative exploratory research design, four focus group interviews were conducted with stakeholders from three Dutch care organizations that have been providing reablement services for at least 6 months prior. Care staff, as well as project leaders, managers, directors, policymakers, and health insurance company representatives, were represented in the interviews. Data were analyzed using the framework method guided by the Consolidated Framework for Implementation Research. In total, 34 stakeholders, distributed over the four focus group interviews, participated in the study. Facilitators included interdisciplinary collaboration, organizational support in terms of leadership engagement and implementation climate, innovation design, and adaptability of the innovation. Barriers included costs and funding of the innovation, time investment (i.e. reporting, number of care visits), and communication. Some factors were listed as both hindering and facilitating such as knowledge and beliefs of care staff, staff’s and clients’ self-efficacy to change, and motivation of clients and family caregivers. These results enhance the understanding of factors influencing the implementation of reablement in community care. The next step is to identify strategies to overcome the identified barriers.

